# Supplementary material for: Survival analysis of pathway activity as a prognostic determinant in breast cancer
Source: PLoS Comput Biol. 2022 Mar 28;18(3):e1010020. doi: 10.1371/journal.pcbi.1010020 (PMC8989354; doi:10.1371/journal.pcbi.1010020)
Supplement: S1 Text — (PDF) [file pcbi.1010020.s001.pdf]

Supporting Information: "Survival analysis of pathway activity as a prognostic determinant in breast cancer"

Gustavo S. Jeuken[1], Nicholas P. Tobin[2], and Lukas Käll[1,*]

[1]Science for Life Laboratory, School of Engineering Sciences in Chemistry, Biotechnology and Health, KTH – Royal Institute of Technology, Solna, Sweden
[2]Department of Oncology and Pathology, Karolinska Institutet and University Hospital, Stockholm, Sweden
[*]Corresponding Author

March 21, 2022

S1 Text: A null model based on set permutation

In the main text, we tested our pathways against the null model that there is a random association between gene expression, and the estimated pathway activity on one hand, and the survival of patients on the other hand. We have shown that our method is well-calibrated under that null.

However, empirical p values can also be calculated against the null hypothesis that the sets of genes are grouped in a pathway are just randomly selected. This is a view that other pathway analysis methods have taken before, e.g. Gene-set enrichment analysis [2, 1].

To test significance under this revised null model, pathway-activities and Cox regressions were evaluated for random gene-sets for all investigated sizes of pathways. These permutations were used to calculate empirical p values. To do this for a pathway of size n, we randomly sample n genes 10 000 times and run our analysis on this collection each time, storing the z values from each regression. This forms the null distribution to which we compare the results of the curated pathway's result again, to derive p values.

We obtained 56 significant pathways at a 5% FDR, which is a contrast to the 1030 pathways found under our main null model, yet provides further confirmation of the significance of our top pathways.

References

[1] Jelle J Goeman and Peter Bühlmann. Analyzing gene expression data in terms of gene sets: methodological issues. *Bioinformatics*, 23(8):980–987, 2007.

[2] Aravind Subramanian, Pablo Tamayo, Vamsi K Mootha, Sayan Mukherjee, Benjamin L Ebert, Michael A Gillette, Amanda Paulovich, Scott L Pomeroy, Todd R Golub, Eric S Lander, et al. Gene set enrichment analysis: a knowledge-based approach for interpreting genome-wide expression profiles. *Proceedings of the National Academy of Sciences*, 102(43):15545–15550, 2005.
