# Supplementary figures and images for: Survival analysis of pathway activity as a prognostic determinant in breast cancer
Source: PLoS Comput Biol. 2022 Mar 28;18(3):e1010020. doi: 10.1371/journal.pcbi.1010020 (PMC8989354; doi:10.1371/journal.pcbi.1010020)

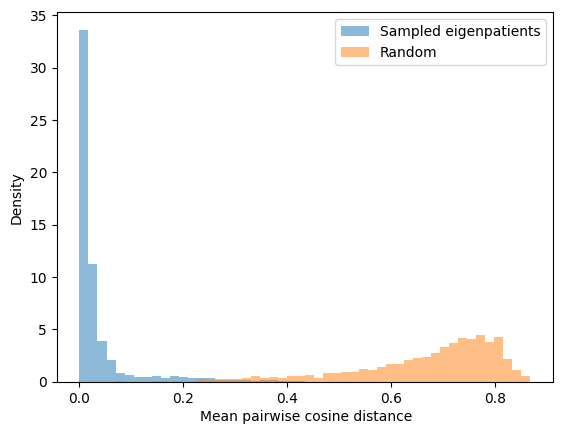

Supplement: S1 Fig — We randomly selected a subset of 20% (398 samples) of the tumors and made pairwise comparisons of the direction of the eigensamples using the cosine distance. To provide a background measure, the procedure was repeated for a collection of random vectors directions picked uniformly in spaces with the same dimensions as the original data. (TIF) [file pcbi.1010020.s002.tif]

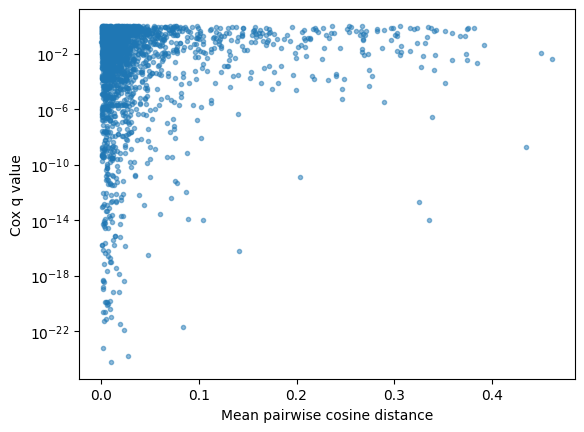

Supplement: S2 Fig — We compare the results of the test of stability against the statistical significance derived from the regression of each pathway’s activity against survival. (TIF) [file pcbi.1010020.s003.tif]

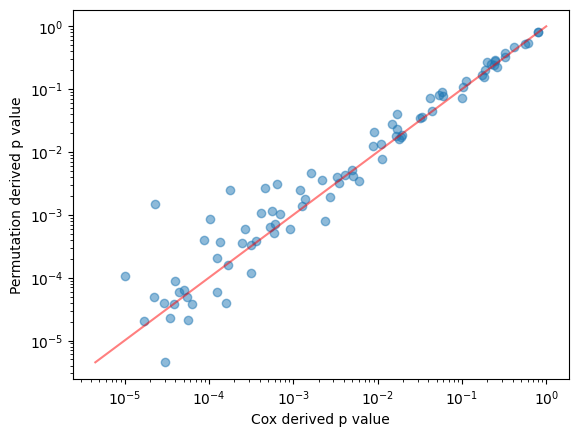

Supplement: S3 Fig — The associations between gene expression values and survival status were permuted and the fraction of permutations with a more extreme outcome was compared to the Cox model’s p value. (TIF) [file pcbi.1010020.s004.tif]

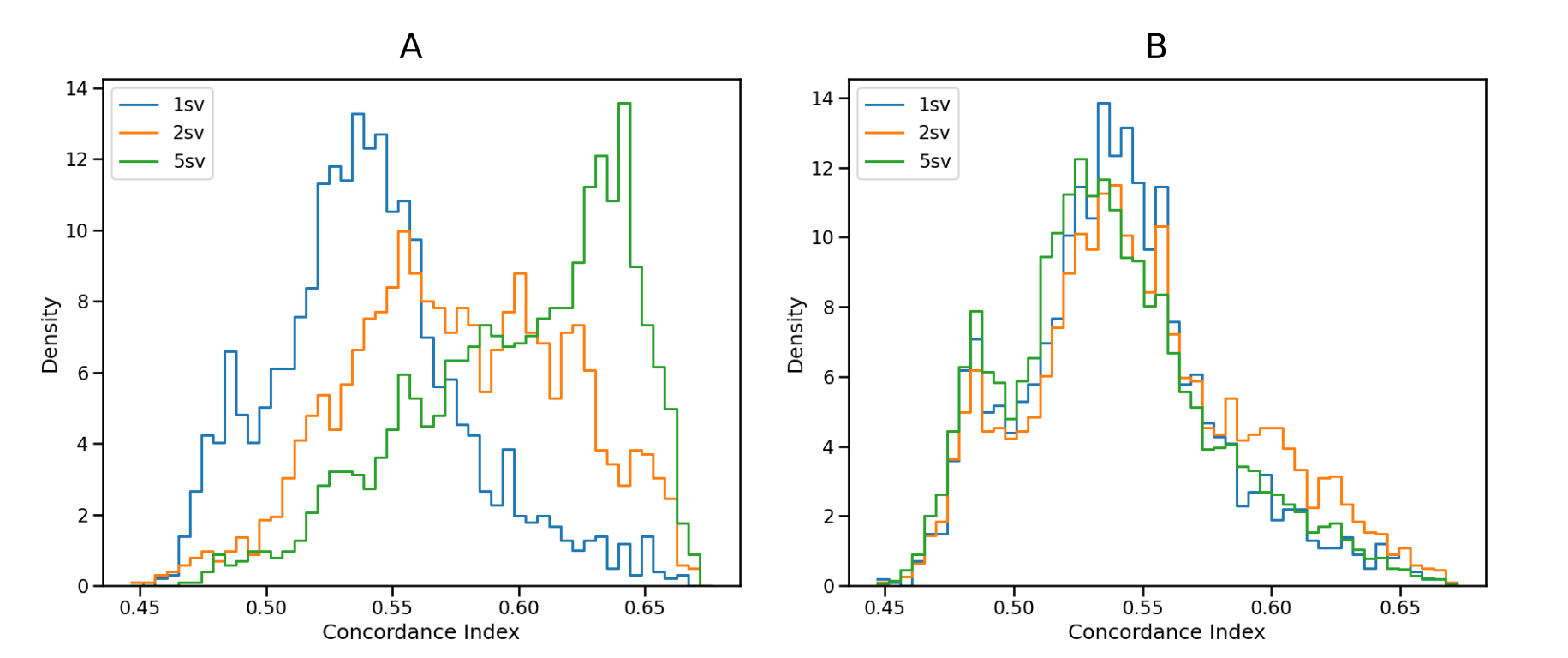

Supplement: S4 Fig — A) The concordance index distribution when building on joint regression model including a specified number of eigenes of the same pathway. B) The concordance index distribution when builing separate regression models for each eigengene. (TIF) [file pcbi.1010020.s005.tif]

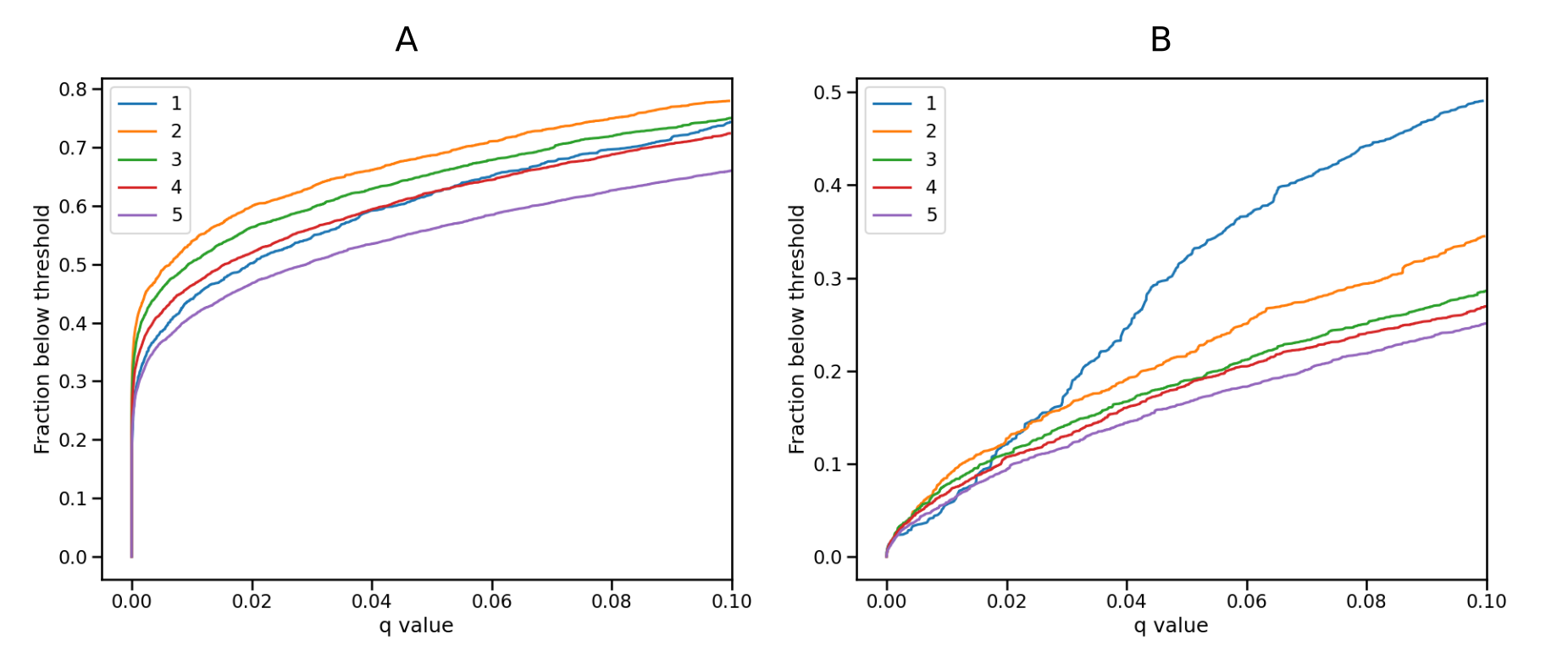

Supplement: S5 Fig — A) The fraction of tests that are under a certain q value threshold, by regressing the n ∈ [1, 5] fist singular vectors of each pathway together, as well as for testing individual transcripts. B) Same context, but now we control for the proliferation signal in each regression model. (TIF) [file pcbi.1010020.s006.tif]
