# Supplementary material for: Survival analysis of pathway activity as a prognostic determinant in breast cancer
Source: PLoS Comput Biol. 2022 Mar 28;18(3):e1010020. doi: 10.1371/journal.pcbi.1010020 (PMC8989354; doi:10.1371/journal.pcbi.1010020)
Supplement: Attachment — Submitted filename: Cover_letter_revised_PCOMPBIOL-D-21-01051.pdf [file pcbi.1010020.s009.pdf]

Dear editor:
Please find attached a revised version of PCOMPBIOL-D-21-01051.
We are thankful for your and the reviewer's constructive comments, which indeed make our manuscript look better. Particularly we are grateful for reviewer 1 pointing out the absence of references to Tomfohr *et al.*, which describes the singular vector decomposition-based method PLAGE, which indeed gives the foundation for our work.

Our revised manuscript is accompanied by a diff from the previous submission so that you can easily compare the versions.

Yours sincerely,
Gustavo Jeuken & Lukas Käll

Reviewer #1:

> *This paper presents an approach to generating biological pathway scores for individual samples and using them for survival analysis. This results in discovery of pathways which are significant predictors of survival. New approaches to pathway analysis are always welcome, especially those which generate single sample scores.*
> *The paper is nicely written and mostly easy to follow. The work seems soundly performed. My main concern is over novelty and significance of the work as detailed below.*
>
> *1. The scoring method seems very similar to another single sample pathway method PLAGE (Tomfohr, J., J. Lu, and T. B. Kepler. 2005., BMC Bioinformatics, 6: 225.). This method also uses SVD to obtain sample level pathway scores. From this it would seem the novelty of the current manuscript derives from coupling the SVD scoring method with conventional survival analysis.*

Here we would like to thank the reviewer for pointing out this flaw in our manuscript. To put it plainly, we had actually had not heard about the PLAGE method before. The revised manuscript gives direct credit to PLAGE for the support vector-based methodology that we use in the manuscript. There is a slight difference, we log-transform our gene expression measures before we apply the SVD-operation which gives us slightly better results than when not log-transforming data.

It should, however, be pointed out that the manuscript is (and was also in its previous incarnation) primarily focused on how to handle the subsequent survival analysis, and how to compensate for the large confounding effect of proliferation in breast cancer.

> *2. It is a little unclear what the exact gap or need is being addressed by this method. In the introduction, it is stated that the technique summarises pathways 'distinctively from the statistical analysis", by which I think they mean the outcome variable is not used to form the pathway scores (ie it is unsupervised). Is this the main objective? There are*

several other unsupervised single sample pathway methods, e.g. ssGSEA, PLAGE, singscore, MOGSA. I would encourage the authors to explain more clearly the motivation for their work.

We have modified the introduction so that it (i) gives a more complete review of existing methods and (2) better reflects our claims for novelty.

3. Line 87 "pathway activities derived previously as independent variables X". Clearly pathways overlap significantly and therefore the pathway scores will not be independent. How is this accounted for?

We agree with the reviewer that a big caveat of our analysis is that we treat pathways as statistically independent. This is an unrealistic assumption, broken not only by the structure of the data (overlapping genes) but also by the fact that gene expression itself is not independent.

While we have shown that each individual test in our model is calibrated under the null hypothesis of a random association between gene expression and survival status, this has strong implications when we discuss false discovery rates, which assumes independent tests.

However, this is a known problem in many established pathway analysis methods, such as GSEA and Over Representation Analysis (ORA), and while there are methods that try to address this by the use of network topology techniques, the reliance on constructed networks comes with its own challenges.

We agree that this is an important problem for this type of analysis, and have added some text in the discussion on the issue. We also hope that by using the hierarchy when visualizing the results on a sunburst pilot, we will draw attention to the lack of interdependence of the results. Yet, ultimately, we feel that the solution to this problem is outside the scope of our manuscript.

Finally, in the specific sentence pointed out, we use the term 'independent variable' in the context of the Cox's regression, where it is standard terminology to denote the regressors this way. We agree that this can be confusing and have changed it to 'explanatory variable' instead.

4. Why do the authors only used the first singular vector to summarise the pathway activity? What proportion of the variance is accounted for? Presumably large pathways may require more singular vectors to account for a similar proportion of variance and therefore the method more accurately summarises small pathways.

When designing the method, we actually did try to use two or more singular vectors for each pathway in our initial analysis. However, the number of tested hypotheses directly scales with the number of included singular vectors, and due to the increased burden of the multiple hypotheses corrections, in effect, the sensitivity drops by including more singular vectors. We did not think this reasoning should be included in our reasoning. We also note that PLAGE only uses the first component, and although it is inadvertent from our side, it makes sense to follow their lead. We also felt that the explained variance was not a good measure of fit, as it indeed correlates so much with the size of each pathway.

5. Equation 3: Please explain in a little more detail, as many readers may not be familiar with this. E.g. what is meant by survival status d? (Is this not the censoring indicator variable?)

The reviewer is right that we were too short in our explanation. In the revised version of the manuscript, we have expanded the section so that it becomes more clear.

6. Stability comparison: The main text says cosine distance was used to compare the direction of subsample eigensamples with original eigensamples. But SI Fig 1 x-axis label is "concordance index". Additionally, it is stated that "the most significant pathways in respect to survival prognosis were all stable". Where is this shown? What effect did the subsampling have on survival prediction (e.g. distribution of concordance index)? The null represented by sampling expression values from U(0,1) seems a bit unrealistic.

We agree with the reviewer that the figure is wrongly labelled, and we changed the x-axis label to "mean pairwise cosine distance", which accurately describes the data. We also added a figure S2 to the supplement, which compares the stability of pathways against the significance derived from the Cox's regression, showing the most significant pathways are stable.

On this specific point in the analysis, we wanted to show that this method of obtaining pathway activities is stable and replicable, using subsampling as a way to simulate repeated experiments, but as we were not looking to study the effects on the regression.

Finally, as the 'mean pairwise cosine distance' is a metric that is hard to put in context, we used a background of randomly sampled vectors to provide a clear reference point. We do not intend to represent gene expression using this sampling method. The text was indeed confusing on this and has been changed to clarify this point.

7. Figure 3: The distributions look bimodal. Do the authors have any insight into why this is?

The plot is likely not bimodal, but we agree that there is a dip near ci=0.5. It is hard to say definitely why that is observed, but an explanation is that the transcription profiles of the analysed samples are so affected by the difference in proliferation, which also affects the survival of the patients. Hence, almost all genes have a small positive or negative component relating to the survival of the patients, which depletes the ci=0.5 region of the plot.

Also, do the best predicting pathways encompass the best-predicting genes?

Earlier versions of the manuscript contained a plot comparing the performance of pathways against their constituent transcripts. This was later removed as we did not find a way of doing this one-to-many comparison in a clear way. Yet, the answer to this question can be clearly stated: The top 10 best predicting pathways contain 6 of the 10 best predicting transcripts, but the presence of such transcripts is not a sufficient condition for a good predictive pathway, as they are also present in many pathways with poor predictive powers. We added this to the text.

8. SI Note 1: The null simulation samples genes independently. Thus the resulting artificial pathways will be much more independent than the real pathways, which is rather unrealistic. In your real pathway set, you might expect more significant pathways since many of them are highly correlated. Instead, you could randomly shuffle the gene-pathway annotation matrix to obtain null pathways which maintain the level of pathway overlap in the real collection.

As noted by the reviewer on point 3, one of the major drawbacks of this approach to pathway analysis is that we treat each pathway independently. This is also true for our null hypothesis based on set permutations. Since the score for each pathway is obtained independently, it is just computationally easier to generate each null distribution separately. This is an approach that is shared with the GSEA gene set permutation method.

9. I could not find the code used to do the analysis.

The code to run the analysis and generate the plots in the manuscript should be in our public repository (https://github.com/statisticalbiotechnology/metabric-pathway-survival). To run it you will need data from both Reactome and the METABRIC consortium. The files from Reactome are freely available and are downloaded when running the script. Unfortunately, we cannot provide the files from the METABRIC consortium, which should be requested directly from them.

Minor comments

10. Several typos or formatting problems, e.g. line 69 “PsubsetG”, lines 145 & 157 “q ! 0.05”. Also, SI figure numbers are missing in the main text.

We have corrected the typos and added the supplementary figure numbers.

11. Figure S2 caption: “associations between gene expression values were permuted”. I think you permuted the correspondence between gene expression values and the survival outcome? Or was each gene independently permuted?

The reviewer is correct in the assumption. We have corrected the text to make this clear.

Reviewer #2:

In this manuscript, Jeuken et al explore the prognostic value of pathway activities based on the transcription profiles of breast cancer of the METABRIC dataset.

I appreciated the paper because well written and for its originality.

I have just some comments to do:

- Authors could better explain if used METABRIC breast cancer dataset overall, or selected patients according to specific criteria;

For this study, the entire METABRIC cohort was used. The consortium responsible for the data reported that there were 12 samples that were reported twice (2012 Curtis et al, supplementary information) and those were removed. We have changed the text to make this clearer.

> *- In addition, the Authors could assess the prognostic value of pathway activities by grouping transcription profiles according to breast cancer subtypes, in order to highlight the influence of the pathways on four different subtypes and their prognosis.*

When we first started to work with this method on the METABRIC dataset, we tried analysing different molecular subtypes of breast cancer separately. We found, however, that proliferation was the strongest signal in terms of survival across all subtypes, and at this point, we decided to study the effects of this signal instead. We also felt that this line of inquiry fits the focus of our manuscript better, as we show the flexibility of using pathway scores.
