# Supplementary material for: Survival analysis of pathway activity as a prognostic determinant in breast cancer
Source: PLoS Comput Biol. 2022 Mar 28;18(3):e1010020. doi: 10.1371/journal.pcbi.1010020 (PMC8989354; doi:10.1371/journal.pcbi.1010020)
Supplement: Attachment — Submitted filename: reply2nd.pdf [file pcbi.1010020.s011.pdf]

Dear editor:

An updated version of our manuscript “Survival analysis of pathway activity as a prognostic determinant in breast cancer” is attached to this letter. The updates are done according to your and the reviewers instructions, but we have also made unprovoked minor clarifications in the text. To facilitate the interpretation of the edits made we have attached a point-to-point reply to the reviewers comments, as well as a version of the manuscript where the differences to the previous manuscript have been highlighted. We hope you find the updates satisfactory.

Yours sincerely,
Gustavo Jeuken, Nick Tobin, and Lukas Käll

Reviewer #1:

This paper presents an approach to generating biological pathway scores for individual samples and using them for survival analysis. This results in discovery of pathways which are significant predictors of survival. New approaches to pathway analysis are always welcome, especially those which generate single sample scores.
The paper is nicely written and mostly easy to follow. The work seems soundly performed. My main concern is over novelty and significance of the work as detailed below.

1. *The scoring method seems very similar to another single sample pathway method PLAGE (Tomfohr, J., J. Lu, and T. B. Kepler. 2005., BMC Bioinformatics, 6: 225.). This method also uses SVD to obtain sample level pathway scores. From this it would seem the novelty of the current manuscript derives from coupling the SVD scoring method with conventional survival analysis.*

Our previous reply: Here we would like to thank the reviewer for pointing out this flaw in our manuscript. To put it plainly, we had actually had not heard about the PLAGE method before. The revised manuscript gives direct credit to PLAGE for the support vector-based methodology that we use in the manuscript. There is a slight difference, we log-transform our gene expression measures before we apply the SVD-operation which gives us slightly better results than when not log-transforming data.

It should, however, be pointed out that the manuscript is (and was also in its previous incarnation) primarily focused on how to handle the subsequent survival analysis, and how to compensate for the large confounding effect of proliferation in breast cancer.

Reviewer #1: Thanks for acknowledging the similarity to PLAGE. The focus of the paper is now clearer, but the result is that the novelty seems less significant.

We would like to thank reviewer #1 for insightful comments. We are glad to hear that the paper is clearer on this aspect now. We also would like to point out that there are several points of our

manuscript that are novel. (i) We study how well SVD based pathway activities can be used for survival analysis. (ii) We are showing how to compensate for the confounding effects of cell proliferation when analysing the survival of patients. (iii) We use sunburst plots for illustration of the hierarchical nature of pathway activities as an aid for exploratory analysis of datasets. To our knowledge, none of these ideas have been put in print previously.

2. *It is a little unclear what the exact gap or need is being addressed by this method. In the introduction, it is stated that the technique summarizes pathways 'distinctively from the statistical analysis", by which I think they mean the outcome variable is not used to form the pathway scores (ie it is unsupervised). Is this the main objective? There are several other unsupervised single sample pathway methods, e.g. ssGSEA, PLAGE, singscore, MOGSA. I would encourage the authors to explain more clearly the motivation for their work.*

Our previous reply: We have modified the introduction so that it (i) gives a more complete review of existing methods and (2) better reflects our claims for novelty.

Reviewer #1: <u>OK thanks for adjusting the introduction</u>.

Thank you.

3. *Line 87 "pathway activities derived previously as independent variables X". Clearly pathways overlap significantly and therefore the pathway scores will not be independent. How is this accounted for?*

Our previous reply: We agree with the reviewer that a big caveat of our analysis is that we treat pathways as statistically independent. This is an unrealistic assumption, broken not only by the structure of the data (overlapping genes) but also by the fact that gene expression itself is not independent.

While we have shown that each individual test in our model is calibrated under the null hypothesis of a random association between gene expression and survival status, this has strong implications when we discuss false discovery rates, which assumes independent tests.

However, this is a known problem in many established pathway analysis methods, such as GSEA and Over Representation Analysis (ORA), and while there are methods that try to address this by the use of network topology techniques, the reliance on constructed networks comes with its own challenges.

We agree that this is an important problem for this type of analysis, and have added some text in the discussion on the issue. We also hope that by using the hierarchy when visualizing the results on a sunburst pilot, we will draw attention to the lack of interdependence of the results. Yet, ultimately, we feel that the solution to this problem is outside the scope of our manuscript.

Finally, in the specific sentence pointed out, we use the term ‘independent variable’ in the context of the Cox’s regression, where it is standard terminology to denote the regressors this way. We agree that this can be confusing and have changed it to ‘explanatory variable’ instead.

Reviewer #1: The authors admit that their method does not take into account correlations among genes and overlaps between pathways. They argue that other methods have similar shortcomings, which is true. The added discussion point is ok. But I would have liked to have seen some attempt at addressing this problem, rather than just pointing out it is a common issue, especially given the lower degree of claimed novelty in the revised manuscript. Finally, I think you mistyped ‘explanatory’ as ‘exploratory’ in the revised text.

We understand the reviewer's desire to stress this point, and we would like to make it clearer that the gene correlation within pathways is taken into account by the scoring method itself. This method was selected specifically because we believe that covariation provides a stronger signal than individual variation.

The issue that remains is gene correlation between pathways. Here, we stress that the only step in our analysis where we make an assumption of statistical independence for pathway scores is when we are calculating the false discovery rates for the same statistics. This will likely result in situations where pathways are deemed regulated due to an overlap with other pathways, but it is not at the core of the analysis we are proposing. We feel it is a problem to be considered on its own, as it is part of most pathway (and individual transcript) analysis methods.

The reviewer is right in that there is a typo in the manuscript, we should have used the term ‘explanatory’ and nothing else. We have now updated the manuscript.

4. Why do the authors only used the first singular vector to summarise the pathway activity? What proportion of the variance is accounted for? Presumably large pathways may require more singular vectors to account for a similar proportion of variance and therefore the method more accurately summarises small pathways.

Our previous reply: When designing the method, we actually did try to use two or more singular vectors for each pathway in our initial analysis. However, the number of tested hypotheses directly scales with the number of included singular vectors, and due to the increased burden of the multiple hypotheses corrections, in effect, the sensitivity drops by including more singular vectors. We did not think this reasoning should be included in our reasoning. We also note that PLAGE only uses the first component, and although it is inadvertent from our side, it makes sense to follow their lead. We also felt that the explained variance was not a good measure of fit, as it indeed correlates so much with the size of each pathway.

Reviewer #1: I appreciate that more singular vectors results in a higher multiple testing penalty. But it seems to me more important to ask whether you are missing information

by only using one singular vector. I think this could have been investigated, or at least commented on.

We modified the manuscript according to the reviewer's suggestion: we introduced a section in the results section investigating the effects of introducing multiple eigenvectors. It turns out that adding extra singular vectors (to a certain amount) improves the results when using the cross validation, yet this is both the result of adding extra degrees of freedom to the regression, and the fact that increasing the number of vectors used exposes us to the risk that one of them captures a strong signal unrelated to the pathway, such as proliferation.

5. *Equation 3: Please explain in a little more detail, as many readers may not be familiar with this. E.g. what is meant by survival status d? (Is this not the censoring indicator variable?)*

Our previous reply: *The reviewer is right that we were too short in our explanation. In the revised version of the manuscript, we have expanded the section so that it becomes more clear.*

Reviewer #1: The extra text is helpful, thanks.

Thank you.

6. *Stability comparison: The main text says cosine distance was used to compare the direction of subsample eigensamples with original eigensamples. But SI Fig 1 x-axis label is "concordance index". Additionally, it is stated that "the most significant pathways in respect to survival prognosis were all stable". Where is this shown? What effect did the subsampling have on survival prediction (e.g. distribution of concordance index)? The null represented by sampling expression values from U(0,1) seems a bit unrealistic.*

Our previous reply: We agree with the reviewer that the figure is wrongly labelled, and we changed the x-axis label to "mean pairwise cosine distance", which accurately describes the data. We also added a figure S2 to the supplement, which compares the stability of pathways against the significance derived from the Cox's regression, showing the most significant pathways are stable.

On this specific point in the analysis, we wanted to show that this method of obtaining pathway activities is stable and replicable, using subsampling as a way to simulate repeated experiments, but as we were not looking to study the effects on the regression.

Finally, as the 'mean pairwise cosine distance' is a metric that is hard to put in context, we used a background of randomly sampled vectors to provide a clear reference point. We do not intend to represent gene expression using this sampling method. The text was indeed confusing on this and has been changed to clarify this point.

Reviewer #1: Thanks for fixing fig S1 and adding fig S2. For fig S2, I think you could use the q-value as the y axis so as the reader can see where the significance level was

drawn. You mention modifying the text to clarify this point, but I could not see where this was. (The whereabouts of changes to the manuscript were not given in the response).

According to the suggestion, we have changed Fig S2's axis from *p* values to *q* values. We would also like to clarify that the small text change was made on the supplement, more specifically on the text explaining the figure.

***7.** Figure 3: The distributions look bimodal. Do the authors have any insight into why this is? Also, do the best predicting pathways encompass the best-predicting genes?*

Our previous reply: The plot is likely not bimodal, but we agree that there is a dip near ci=0.5. It is hard to say definitely why that is observed, but an explanation is that the transcription profiles of the analysed samples are so affected by the difference in proliferation, which also affects the survival of the patients. Hence, almost all genes have a small positive or negative component relating to the survival of the patients, which depletes the ci=0.5 region of the plot.

Earlier versions of the manuscript contained a plot comparing the performance of pathways against their constituent transcripts. This was later removed as we did not find a way of doing this one-to-many comparison in a clear way. Yet, the answer to this question can be clearly stated: The top 10 best predicting pathways contain 6 of the 10 best predicting transcripts, but the presence of such transcripts is not a sufficient condition for a good predictive pathway, as they are also present in many pathways with poor predictive powers. We added this to the text.

Reviewer #1: I think it could be useful to add a short comment on the bimodality to the text. The additional text regarding best-predicting pathways/genes is welcome.

We have added a comment on the bimodality of the distribution to the Results section "It is also interesting to note that in both cases there is a depletion of scores around the null prediction of 0.5, this deviation is further indication that transcripts, as well as pathway scores, are not independent. ".

***8.** SI Note 1: The null simulation samples genes independently. Thus the resulting artificial pathways will be much more independent than the real pathways, which is rather unrealistic. In your real pathway set, you might expect more significant pathways since many of them are highly correlated. Instead, you could randomly shuffle the gene-pathway annotation matrix to obtain null pathways which maintain the level of pathway overlap in the real collection.*

Our previous reply: As noted by the reviewer on point 3, one of the major drawbacks of this approach to pathway analysis is that we treat each pathway independently. This is also true for our null hypothesis based on set permutations. Since the score for each pathway is obtained independently, it is just computationally easier to generate each null distribution separately. This is an approach that is shared with the GSEA gene set permutation method.

Reviewer #1: The authors seem to say that this point does not matter because a) GSEA also has this problem and b) it is computationally easier. I don't find this a very strong argument.

Following our discussion of point 3, we stress that we only use the assumption of independence between pathways when calculating the false discovery rates and that the calculation of pathway scores does not need this assumption.

9. *I could not find the code used to do the analysis.*

Our previous reply: The code to run the analysis and generate the plots in the manuscript should be in our public repository (https://github.com/statisticalbiotechnology/metabric-pathway-survival). To run it you will need data from both Reactome and the METABRIC consortium. The files from Reactome are freely available and are downloaded when running the script. Unfortunately, we cannot provide the files from the METABRIC consortium, which should be requested directly from them.

Reviewer #1: Although the github site is given in the text, nowhere does it say that the code is available there. I think this should be explicitly stated.

We have added a subsection to the Methods, where we describe code availability.

Minor comments

10. *Several typos or formatting problems, e.g. line 69 "PsubsetG", lines 145 & 157 "q ! 0.05". Also, SI figure numbers are missing in the main text.*

Our previous reply: We have corrected the typos and added the supplementary figure numbers.

Reviewer #1: Ok

11. Figure S2 caption: "associations between gene expression values were permuted". I think you permuted the correspondence between gene expression values and the survival outcome? Or was each gene independently permuted?

Our previous reply: The reviewer is correct in the assumption. We have corrected the text to make this clear.

Reviewer #1: Ok

Reviewer #3:

1. *Based on the current results and manuscript, it is hard to justify whether using pathway activities can provide better prognostic value compared to transcript-based evaluation. Also, gene set enrichment analysis (GSEA) of transcriptomes has been used to define a set of genes as a prognostic tool. What is major difference and advantage of this*

pathway-based tool compared to GSEA? Authors may need to discuss differences among different transcriptome analysis strategies and then specifically address why pathway-based tool is relatively precise to evaluate patients' survival time.

We have presented a comprehensive analysis of a method to perform survival analysis based on pathways. As far as we know, this is a very undeveloped area of research. GSEA can be used for comparing pathway activities between patient groups, e.g. case and control, however, it does not handle regressions of censored data, like we do here with the patients' lifetimes. Although this issue is not the main point of our manuscript, we have updated the discussion and introduction to better outline the difference between single-transcript and agglomerated pathway analysis.

2. *Cross-talk between two pathways contributing to tumorigenesis has been considered an important oncogenic event. How can authors deal with this issue using statistical models and whether it will affect the final finding and can be applied as a prognostic tool also.?*

This is a fascinating topic that the reviewer raises. We have not considered this in our manuscript, and we think it goes beyond its scope. We note that having results presented at a pathway-level will facilitate the type of analysis the reviewer suggests. However, we also see a problem in that most pathway databases include non-pathway-unique biomolecules that are included in multiple pathways. Pathways including such biomolecules naturally stand a high risk of correlating in their activity level, just from their definitions.
