# Supplementary material for: Survival analysis of pathway activity as a prognostic determinant in breast cancer
Source: PLoS Comput Biol. 2022 Mar 28;18(3):e1010020. doi: 10.1371/journal.pcbi.1010020 (PMC8989354; doi:10.1371/journal.pcbi.1010020)
Supplement: Attachment — Submitted filename: PLoSComputBiol_Jeuken et al.docx [file pcbi.1010020.s010.docx]

**RE: Survival analysis of pathway activity as a prognostic determinant in breast cancer**

Using the most significant cancer-specific transcript or gene sets as a prognostic tool for evaluating therapeutic efficacy and patients’ survival time has been widely applied in diverse cancer models, including breast cancers. But, Jeuken et al in this study demonstrated that, instead of focusing on transcripts, interrogation of pathway activities gives better prognostic value of evaluating patients’ survival length by analyzing transcriptome profiles of breast cancer of the METABRIC dataset. However, some questions need to be further clarified to support the statements.

Major comments:

1. Based on the current results and manuscript, it is hard to justify whether using pathway activities can provide better prognostic value compared to transcript-based evaluation. Also, gene set enrichment analysis (GSEA) of transcriptomes has been used to define a set of genes as a prognostic tool. What is major difference and advantage of this pathway-based tool compared to GSEA? Authors may need to discuss differences among different transcriptome analysis strategies and then specifically address why pathway-based tool is relatively precise to evaluate patients’ survival time.
2. Cross-talk between two pathways contributing to tumorigenesis has been considered an important oncogenic event. How can authors deal with this issue using statistical models and whether it will affect the final finding and can be applied as a prognostic tool also.?
